# *Citrobacter braakii* Isolated from Salami and Soft Cheese: An Emerging Food Safety Hazard?

**DOI:** 10.3390/foods14111887

**Published:** 2025-05-26

**Authors:** Frédérique Pasquali, Cecilia Crippa, Alex Lucchi, Santolo Francati, Maria Luisa Dindo, Gerardo Manfreda

**Affiliations:** Department of Agricultural and Food Sciences, Alma Mater Studiorum—University of Bologna, 40127 Bologna, Italy; frederique.pasquali@unibo.it (F.P.); alex.lucchi3@unibo.it (A.L.); santolo.francati2@unibo.it (S.F.); marialuisa.dindo@unibo.it (M.L.D.); gerardo.manfreda@unibo.it (G.M.)

**Keywords:** *Citrobacter braakii*, artisanal production, food of animal origin, genomic characterization, *Galleria mellonella* larvae, in vivo infection model, pathogenicity assessment

## Abstract

*Citrobacter braakii* can colonize the intestinal tract of humans and animals and occasionally act as opportunistic pathogen. Although isolated from food and the environment, its potential as a foodborne pathogen remains uncertain. Twenty *C. braakii* isolates were previously collected from salami and soft cheese artisanal productions. In the present study, the potentialities of *C. braakii* as a food safety hazard were explored by a genomic comparison of *C. braakii* newly sequenced genomes with publicly available genomes, including those of clinical relevance, and a pathogenicity assessment in *Galleria mellonella* as an in vivo infection model. Phylogenomic reconstruction revealed that one salami clone and two *C. braakii* genomes of the soft cheese production were closely related (from 11 to 28 core SNP differences) to *C. braakii* publicly available clinical genomes. All genomes carried the chromosomally located *bla_CMY_* and/or *qnrB* genes and were resistant to cephalosporins and/or had reduced susceptibility to ciprofloxacin. *G. mellonella* larvae showed 90% mortality after challenge with *C. braakii* strains carrying the *vex* and *tvi* operons coding for the capsular polysaccharide (Vi antigen), in comparison to 40% of strains lacking these two operons. The high mortality rate of *vex*- and *tvi*-positive *C. braakii* isolated from food processing plants suggests *C. braakii* to be a possible foodborne hazard.

## 1. Introduction

*Citrobacter* spp. are facultative anaerobic bacteria belonging to the *Enterobacteriaceae* family. As commensal inhabitants of the intestinal tract, they can colonize both humans and animals. They are also recovered from the environment and can act as opportunistic pathogens in humans. Infections due to *Citrobacter* species are increasingly being observed in hospitalized patients and are often multidrug-resistant [1,2,3,4,5].

Among *Citrobacter* spp., *C. freundii* is the most frequently identified species in nosocomial infections, whereas *C. braakii* comprises 10–20% of all *Citrobacter* spp.-associated diseases [2]. The low isolation rate of *C. braakii* might be associated with frequent misidentification. *C. braakii* shares phenotypic features with *C. freundii* and other members of the *Enterobacteriaceae* family, such as *Escherichia coli* and *Salmonella*. The biochemical similarities it shares other bacterial species lead to inaccurate diagnosis by traditional phenotypic methods [6,7,8,9,10,11].

At present, the literature related to the isolation and foodborne transmission of *Citrobacter* spp. is primarily focused on *C. freundii* [12]. However, the misidentification of *C. braakii* might be responsible for it being underreported, ultimately leading to the relatively low prevalence of *C. braakii* in clinical settings. The application of alternative identification approaches with high resolution, such as whole-genome sequencing, might lead to increased identification, potentially revealing *C. braakii* as a future emerging pathogen. At present, little information is available on *C. braakii*, specifically in relation to its potential as a foodborne hazard.

In humans, *C. braakii* has been linked to bloodstream infections, green nail syndrome and bacteremia, associated or not with septic shock [6,9,13,14,15]. Its proinflammatory and cytotoxic role in gastric epithelial cells has also been described [16]. Information on the virulence of *C. braakii* is limited to a cytotoxic strain isolated from the human stomach. In this strain, the type 6 secretion system (T6SS) and the adhesion-related *fim* and *csg* gene clusters were described, although the authors did not investigate whether these genes were key virulence markers associated with clinical manifestations [16].

Apart from humans, *C. braakii* has occasionally been found in wastewater, meat and meat products, ready-to-eat food, feed of animal origin, and the intestine and faeces of animals such as fish [10,11,17,18,19,20,21,22]. Different to *Citrobacter freundii*, which has been frequently described as a foodborne pathogen [12], at present, there is a lack of data on *C. braakii* associated with foodborne diseases, although it has been identified in food products, food-producing animals and diseased humans.

Within an EU-funded project named ARTISANEFOOD in which the microbiological hygiene of artisanal cheeses and salami productions of different European countries were compared, 1170 samples were previously collected in 6 batches of 1 soft cheese and 1 salami artisanal production from January 2020 to May 2021. Samples from the processing environment, raw materials, and semi-finished and finished products were collected in order to assess the impact of non-fully automated processing on the variability of the microbiological quality of food of animal origin in consecutive batches [23,24]. In this context, *Enterobacteriaceae* isolates were characterized at the species level by biotyping, revealing the presence, among others, of *Citrobacter freundii* and *Klebsiella* spp. [23,24]. *Klebsiella* spp. isolates were previously sequenced and included in a published study that focused on the investigation of the virulome of their newly sequenced genomes in comparison to publicly available genomes from humans and pigs [25]. Two of these biotyped *Klebsiella* were reattributed to *C. braakii* after sequencing and discarded from the previous study [25]. In the present study, 19 biotyped *C. freundii* were sequenced and 18 of them were confirmed as belonging to *C. braakii*. The aim of the present study was to characterize, at genome level, 20 *C. braakii* previously collected from artisanal food of animal origin in order to gain insights on their virulence patterns, pathogenicity potential and transmission. In particular, this study aims to (1) explore the genetic relationships among newly sequenced foodborne genomes and publicly available clinical genomes; (2) characterize their resistome and virulome; and (3) assess their pathogenicity by using *Galleria mellonella* as an in vivo infection model.

## 2. Materials and Methods

### 2.1. Rationale for Isolate Selection

In a previous study, 1170 samples were collected from raw materials, semi-finished and finished products and the processing environment from six production batches from January 2020 to May 2021 in two artisanal productions of soft cheese and organic salami [23,24]. After its identification by biotyping, *C. freundii* was detected in 2 samples of soft cheese finished products and in 17 samples of the salami production, namely from raw materials, the processing environment (i.e., tables and filler stuffers) and semi-finished products at 18 weeks of ripening (Table 1). Along with *C. freundii*, biotyping previously revealed 75 isolates of *K. pneumoniae* and *K. oxytoca* [25]. Among *Klebsiella*, isolates 6CP485A and 6STM5 were biotyped as *K. pneumoniae* collected from a sample of finished cheese and *K. oxytoca* collected from the salami processing environment, respectively. These two *Klebsiella* spp. isolates were subsequently sequenced and confirmed as belonging to *C. braakii* (and were therefore discarded in a previous study focused on *Klebsiella* spp.) (Table 1) [25]. Due to the potential misidentification of *C. braakii* by phenotypic methods [6,7,8,9,10,11], all 19 *C. freundii* isolates were subjected to sequencing in the present study for species confirmations.

### 2.2. DNA Extraction and Whole-Genome Sequencing (WGS)

Isolates were grown overnight at 37 °C on Brain Heart Infusion broth (Thermo Scientific™, Waltham, MA, USA) and then submitted to DNA extraction for sequencing purposes using the MagAttract HMW DNA Kit (Qiagen, Hilden, Germany), according to the manufacturer’s instructions. BioSpectrometer fluorescence (Eppendorf, Milan, Italy) was used to measure the purified DNA concentration and the quality parameter ratio 260/280. The whole genome of isolates was paired-end sequenced (2 × 150 bp) using the Illumina NovaSeq platform (Illumina, Milan, Italy). After genome sequencing, raw reads were de novo assembled using Unicycler v0.5.0 [26], and the quality of the assembled contigs was evaluated with contig_info v2.01 [27].

### 2.3. Sequence-Based Taxonomic Assignment and Sequence Type Definition

In order to avoid possible misidentification by biochemical tests, strains’ taxonomy at the species level was confirmed with ReferenceSeeker v1.8.0 [28] by matching genomic sequences with closely related reference genomes hosted in the RefSeq pre-built database [29]. Confirmed strains were then analysed with mlst v2.23.0 [30] for sequence type (ST) definitions based on the PubMLST typing scheme [31]. *Citrobacter* spp. isolates with unidentified STs were submitted to the pubMLST.org platform for new alleles/ST assignment.

### 2.4. Phylogenetic Analysis by SNP Calling

Phylogenetic inference was performed using a core SNP-based approach. Publicly available genomes were retrieved to complement the newly sequenced *Citrobacter* spp. isolates. A total of 263 *C. braakii* assemblies were initially collected from the NCBI and PubMLST databases [31,32]. For the assemblies downloaded from the NCBI, metadata were retrieved using the NCBImeta tool v0.8.3 [33], whereas PubMLST assemblies already had metadata available on the server. Assemblies were selected based on source information, retaining only isolates derived from human, animal, or environmental origins. Genomes lacking source metadata or assigned to other sources were excluded from further analyses.

Quality filtering was subsequently performed using the contig_info v2.01 pipeline [27]. Assemblies were retained if they satisfied the following criteria: genome size between 4.5 and 6.5 Mb, GC content between 52% and 54%, fewer than 1000 contigs, and N50 values greater than 20,000 bp. Assemblies not meeting these thresholds were excluded. Assembly quality metrics and metadata are summarized in Table S1.

Core SNPs were identified using kSNP4.1, with the optimum k-mer size (k = 17) determined by Kchooser4 [34]. A maximum likelihood (ML) phylogenetic tree was generated based on the core SNPs. While kSNP4.1 does not allow the manual selection of the nucleotide substitution model, the analysis is generally based on a standard substitution model commonly used in ML-based phylogenetic inference (e.g., GTR-like models).

The tree was rooted using the *C. freundii* genome (strain 6CP485A) included in the dataset as an outgroup. Clade designation was based on monophyletic clusters in the ML tree and supported by metadata (source and strain origin). The tree was visualized and annotated with strain metadata using iTOL v6 [35], and a pairwise SNP distance matrix was computed using snp-dists v0.6.3 [36]. As kSNP4.1 does not support the calculation of bootstrap or support values, topological confidence was not reported.

In addition, to investigate gene content variation among strains, presence/absence matrices of selected virulence and resistance genes were generated by ABRicate v1.0.1 [37] using Resfinder and VFDB databases, respectively (Tables S2 and S3). The key features of the localization and spread of virulence and antimicrobial resistance were visualized with SnapGene Viewer v7.1.1 [38].

### 2.5. Prediction of Resistance and Virulence Determinants and Mobilization

*Citrobacter* spp. contigs were screened for antimicrobial resistance- and virulence-associated genes by ABRicate v1.0.1 [37] using the Resfinder and VFDB databases, respectively. Moreover, chromosomal point mutations associated with antimicrobial resistance were identified using the web tool ResFinder v4.6.0 [39]. The location of both antimicrobial resistance and virulence genes was predicted using MOB-suite v3.1.9 [40]. To evaluate whether the prevalence of potential key virulence markers differed between *Citrobacter* spp. isolated from different sources, statistical analyses were performed in R v4.3.2. In particular, the presence of *vexABCDE* and *tviBCDE* operons coding for a capsular polysaccharide (Vi antigen) was compared between publicly available isolates of human and environmental origin using Pearson’s chi-square test (χ^2^) as all expected cell counts were ≥ 5. The expected counts were calculated using the default method implemented in R’s chisq.test () function, based on marginal totals under the assumption of independence. Moreover, the presence of adhesion-related *fim* and *csg* gene clusters was also compared between human and environmental isolates by using Fisher’s exact test due to the presence of expected cell counts < 5. These genes were selected for analysis as potential key virulence markers of *C. braakii* for its pathogenicity in humans. For each comparison, an odds ratio (OR) with a 95% confidence interval (CI) was calculated to quantify the strength of the association. No correction for multiple testing was applied, given the limited number of comparisons and the use of a conservative significance threshold. Statistical significance was determined using a threshold of α = 0.001.

### 2.6. Antimicrobial Susceptibility Testing

Following the detection of the *bla_CMY_* and *qnrB* genes, Sensititre™ plates (Thermo Scientific, Waltham, MA, USA) were used according to the gold-standard broth microdilution phenotypic assay [41] in order to phenotypically confirm the predicted resistance against AmpC β-lactams and reduced susceptibility to fluoroquinolones. Isolates were tested against ciprofloxacin (CIP), cefotaxime (FOT), ceftazidime (TAZ), cefoxitin (FOX), cefepime (FEP), cefotaxime/clavulanic acid (F/C), ceftazidime/clavulanic acid (T/C), and temocillin (TRM). The isolates were defined as susceptible or resistant according to the clinical breakpoints (CBPs) established by the European Committee on Antimicrobial Susceptibility Testing (EUCAST) [42]. *Escherichia coli* ATCC25922 was included as a quality control strain in each batch of analyses.

### 2.7. Pathogenicity Assessment

A laboratory colony of *Galleria mellonella* was maintained at the entomology unit of the Department of Agricultural and Food Sciences of the Alma Mater Studiorum—University of Bologna. The larvae were kept in plastic boxes (24 cm × 8 cm × 8 cm), maintained at 30 ± 1 °C, 65 ± 5% RH, 0:24 L:D photoperiod, and reared on an artificial diet, which was supplied three times a week [43]. For the experiment, last instar larvae were used and infected, as previously described by Gallorini and colleagues [44]. Briefly, selected strains were cultured on Mueller Hinton Agar plates (MHA, Thermo Scientific™, Waltham, MA, USA) at 37 °C in aerobic conditions. Bacteria were transferred in 8 mL of Mueller Hinton II Broth (MH2B; Sigma-Aldrich, Milan, Italy) and incubated at 37 °C and 125 rpm in aerobiosis. After 16 h of incubation, bacteria were harvested by centrifugation at 10.000 rpm for 5 min at 4 °C. The supernatant was discharged, and the cellular pellet was washed in Phosphate-Buffered Saline (PBS; Sigma Aldrich, Milan, Italy), followed by another step of centrifugation as before. Bacterial cells were re-suspended in PBS, and the optical density was measured at 600 nm (OD600) to obtain the proper bacterial suspension. Larvae weighing within 200–250 mg were selected. Each administration required the injection of a volume corresponding to 10 μL in the third left pro-leg of the larva. Approximately 10^6^ CFU/larva was the infection dose as previously described [45]. After dilutions, the infection dose was evaluated by plating 100 µL of each of serial dilution on Brain Heart Infusion agar (Thermo Scientific, Waltham, MA, USA). After incubation at 37 °C overnight, the infection dose was determined as ranging from 5.71 to 5.95 log10 CFU/mL. Four strains were selected based on their virulence patterns, and each strain was administered in ten larvae in triplicate. PBS was inoculated in ten larvae in triplicate as a negative control. Additionally, ten larvae in triplicate were not injected to control for background larval mortality. A total of 180 larvae were included in the experiment. After infection, larvae were stored in the dark at 35 °C, and the survival rate was assessed daily over 5 days. Survival curves were generated using the Kaplan–Meier method, and statistical differences among groups were assessed by the log-rank test. Analyses were performed using the survival package in R v4.3.2. A *p*-value < 0.05 was considered statistically significant.

## 3. Results

### 3.1. Citrobacter Prevalence and Taxonomic Assignment Across Food Processing Facilities

Following whole-genome sequencing, biotyped *Klebsiella* spp. isolate 6CP485A was reassigned to *C. freundii*, whereas 18 biotyped *C. freundii* and *Klebsiella* spp. isolate 6STM5 were reassigned to *C. braakii*. All *C. braakii* genomes shared percentages of Average Nucleotide Identity (ANI) between 99.04% and 99.40% (Table 1). Overall, the prevalence of *C. braakii* in artisanal food processing facilities was 1.7%.

### 3.2. De Novo Assembly

Draft genomes showed excellent quality statistics: a small number of contigs (38–232), high N50 (99247–2641110) and the largest contig size (from 231,340 to 2,641,110 bp). Genome length (4.8–5.6 Mb) and GC contents (51.0–52.2%) were in the typical range for *Citrobacter* spp. (Table S4).

### 3.3. Phylogenetic Analyses and Sequence Type Distribution

**Figure 1:**
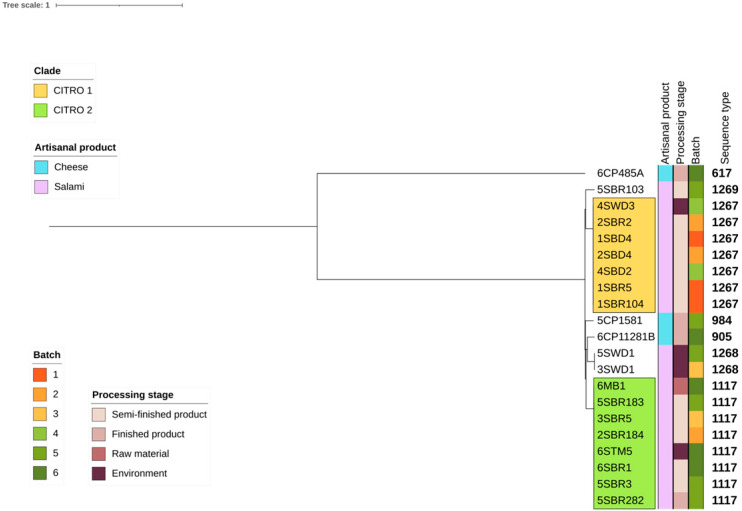
Maximum likelihood phylogenetic tree inferred from core gene alignments of 20 *C. braakii* and 1 *C. freundii* newly sequenced genomes isolated from two Italian salami and cheese artisanal productions. The tree was rooted with the *C. freundii* genome (6CP485A). Clusters CITRO 1 (yellow) and CITRO 2 (green) are indicated.

MLST analyses pointed out four known (ST617, ST905, ST984 and ST1117) and three newly assigned (ST1267, ST1268 and ST1269) sequence types, resulting in seven different STs identified (Figure 1, Table S5). In order to investigate the genetic relationship among newly sequenced genomes and potential routes of transmission within the food processing plant, a maximum likelihood (ML) phylogenetic tree was inferred from the SNP calling. The phylogenomic reconstruction shaped two distinct major clades, namely CITRO1 and CITRO2, which both gathered isolates of the salami production harbouring the same STs (ST1267 and ST1117, respectively) (Figure 1). CITRO 1 gathered seven genomes of *C. braakii* with the core SNP differences included ranging from zero to three, suggesting a clonal relationship among the isolates (Table S6). In particular, four out of seven genomes belonged to *C. braakii* isolated from the drying room either from the environment (drains) (4SWD3) or from semi-finished products (2SBD4, 1SBD4, and 4SBD2), suggesting the environment of the drying room to be a potential source of salami contamination. CITRO 2 gathered eight closely related genomes of *C. braakii* with core SNP differences ranging from zero to one (Table S6). Within CITRO 2, one *C. braakii* was isolated from raw materials (a mixture of pig meat and spices) (6MB1), one from the surface of the table in the stuffing room (6STM5), five from semi-finished salami (5SBR183, 3SBR5, 2 SBR184, 6SBR1, and 5SBR3) and one from a finished product (5SBR282) collected in the ripening room. These results suggest the entrance of *C. braakii* of CITRO 2 in the facility through contaminated raw materials, and the spread of contamination along the whole production chain up to the final product. In both clades, closely related genomes were collected from different batches, suggesting the potential persistence of two different clones during subsequent batches.

**Figure 2:**
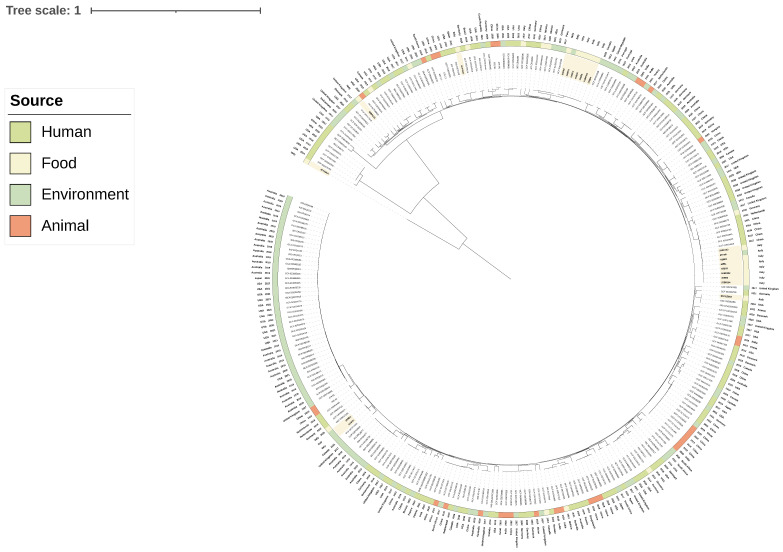
Maximum likelihood phylogenetic tree inferred from core gene alignments of one *C. freundii* and 284 *C. braakii* including the 21 newly sequenced genomes and 263 publicly available genomes of human, food, animal and environmental origin. The tree was rooted with the *C. freundii* genome (6CP485A). Close genetic relationships between clinical and newly sequenced *C. braakii* genomes are represented by nodes displayed in bold red.

In order to decipher the genetic relationship of the newly sequenced *C. braakii* with isolates collected previously in diseased humans and food, animal, and environmental sources all over the world, a maximum likelihood (ML) phylogenetic tree was inferred including 263 publicly available genomes, along with the 21 genomes of the present study (Figure 2). Interestingly, genomes of clade CITRO 2 from salami production showed a close genetic relationship (from 26 to 28 core SNP differences) with *C. braakii* genomes isolated from humans in China and The Netherland (Figure 2, Table S6). Moreover, two other clusters with *C. braakii* strains isolated from soft cheese displayed high similarity with human genomes: 6CP11281B and human genomes from the USA and France (14 and 15 core SNP differences) and 5CP1581 with human genomes from Spain and Germany (11 and 27 core SNP differences) (Figure 2, Table S6). Despite these low SNP differences, the isolates were recovered in different years and countries, and no epidemiological link could be established. Thus, these findings suggest a recent common ancestry rather than direct transmission events, supporting the hypothesis that foodborne *C. braakii* strains may act as potential opportunistic pathogens for humans.

### 3.4. Resistome and Antimicrobial Susceptibility Testing

**Figure 3:**
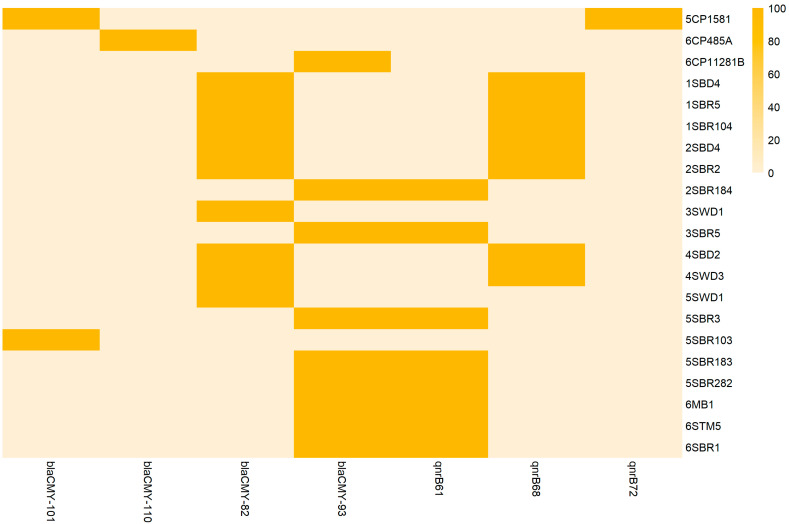
Heatmap of the resistome of 20 newly sequenced *C. braakii* genomes and 1 *C. freundii* genome (6CP485A). Values are scaled from 0 to 100, representing percentage identity with reference AMR gene sequences.

Based on whole-genome sequencing, the antimicrobial resistance of *C. braakii* isolates was predicted. All newly sequenced genomes carried the *bla_CMY_* gene either alone or in combination with the *qnrB* gene, predicting resistance to *AmpC* β-lactams and reduced susceptibility to fluoroquinolones (Figure 3). In particular, *bla_CMY-82_* and *qnrB68* genes were found in CITRO1 cluster strains; *bla_CMY-82_* alone in 3SWD1 and 5SWD1 strains; *bla_CMY-93_* and *qnrB68* in CITRO 2 cluster strains; *bla_CMY-93_* alone in 6CP11281B; *bla_CMY-101_* and *qnrB72* genes in 5CP1581; and *bla_CMY-101_* alone in the 5SBR103 strain. All *bla_CMY_* and *qnrB* gene variants were chromosomally located in separated genetic environments already reported in published genomes of *C. freundii* [46,47,48,49]. In particular *bla*_CMY_ was surrounded by the *sugE* and *blc* genes upstream and the *ampR* gene downstream, whereas the *qnrB* gene was surrounded by the *pspFABCD* operon upstream and the *sapABCDF* operon downstream.

AMR genes in newly sequenced genomes and publicly available genomes were compared. The *bla_CMY_* gene was detected in all food (including the newly sequenced ones) and animal genomes, in all but one environmental genomes and in all but six human genomes (Table S2). The *bla_CMY_* gene variants detected were *bla_CMY_* 2-6-16-70-74-82-83-93-100-101 (Table S2). This gene was found in 97.9% of the genomes, suggesting the wide distribution and stability of this gene among *C. braakii* genomes, probably also due to its chromosomal location. The *qnrB* gene was less represented, with 49% of the genomes being positive, irrespective of the source. The *qnrB* gene variants detected were qnrB1-2-4-6-10-19-27-40-51-61-67-68-70-71-72 (Table S2).

MIC values higher than 8 mg/L of cefoxitin were found in 16 out of 20 *bla_CMY_*-positive isolates. One *bla_CMY-93_*-positive isolate (6STM5) was susceptible to cefoxitin and resistant to cefotaxime, ceftazidime, a combination of both with clavulanic acid and temocillin (Table S7). Three *bla_CMY_*-positive isolates (3SWD1, 5SWD1 and 6STM5) were susceptible to all tested antimicrobials including β-lactams. The genotype to phenotype discordance for these three isolates requires further investigations. In particular, the downregulation of *AmpR* has been previously associated with a reduced expression of AmpC β-lactamase-encoding genes in *Citrobacter freundii. ampR* downregulation has been previously associated with point mutations [50]. In the three newly sequenced genomes 3SWD1, 5SWD1 and 6STM5, the *ampR* gene showed a homology of only 92% in comparison to the same gene of the *C. freundii* reference strain (NZ_CP033744.1) (Table S8). Further analyses should be performed to confirm the association of the identified point mutations with the gene downregulation. Regarding fluoroquinolones, MIC values of ciprofloxacin were below 0.015 for *qnrB*-negative isolates and ranged from 0.03 to 0.25 for *qnrB*-positive isolates, confirming the predicted reduced susceptibility of the latter.

### 3.5. Virulome and Pathogenicity Assessment

**Figure 4:**
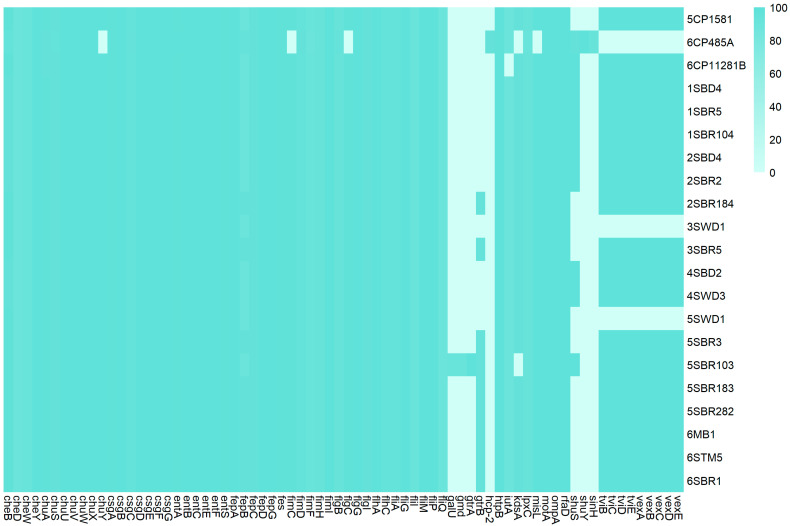
A heatmap of the virulome of 20 newly sequenced *C. braakii* genomes and 1 *C. freundii* genome (6CP485A).

The investigation of virulence-associated genes in newly sequenced *C. braakii* genomes revealed similar patterns, with the number of virulence gene orthologs ranging from 54 to 65 (Figure 4). Based on the heatmap of the virulome, newly sequenced genomes can be gathered in four virulence clusters: (1) *galU*-, *gmd*-, *gtrA*-, *gtrB*-, *shuS*-, *tvi*- and *vex*-negative; (2) *shuS*-, *tvi*- and *vex*-positive; *galU*-, *gmd*-, *gtrA*- and *grtB*-negative; (3) *gtrB*-, *tvi*- and *vex*-positive; *galU*-, *gmd*- and *gtrA*-negative; and (4) *galU*-, *gmd*-, *gtrA*-, *gtrB*-, *shuS*-, *tvi*- and *vex*-positive. In order to assess the pathogenicity of newly sequenced *C. braakii*, *Galleria mellonella* larvae were tested as an in vivo infection model. Four isolates representative of each clade and virulence cluster were selected for the in vivo infection experiments: 1SBR104 (belonging to clade CITRO 1, virulence cluster 2), 5SBR282 and 5SBR103 (both belonging to CITRO 2 and virulence clusters 3 and 4, respectively) and 3SWD1 (virulence cluster 1) (Figure 1 and Figure 4).

**Figure 5:**
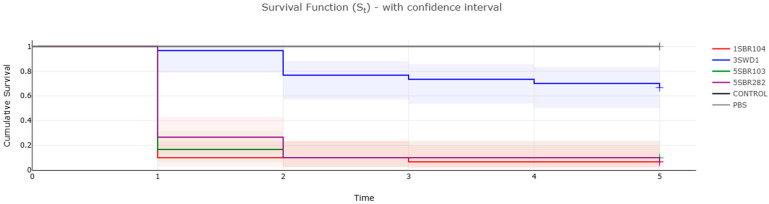
Kaplan–Meier plot showing percentage survival of *Galleria mellonella* larvae after inoculation with bacterial suspensions of *C. braakii* strains representative of identified virulence patterns. Non-injected larvae (CONTROL) and larvae injected with sterile PBS (PBS) are included. For each treatment, *n* = 30 (pooled from triplicate experiment).

**Figure 6:**
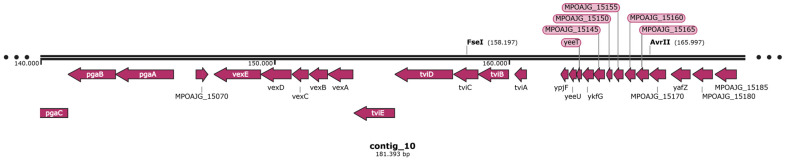
Genetic surroundings of *C. braakii* strain 2SBR2 carrying *tvi*BCDE and *vex*ABCDE operons. Each arrow represents a gene, with the arrowhead indicating the direction of trascription.

Survival curves in the Kaplan–Meier analysis revealed the higher pathogenicity of isolates 1SBR104, 5SBR103 and 5SBR282 in comparison to the isolate 3SWD1 (Figure 5). The three isolates were associated with a mortality of infected *G. mellonella* equal to or higher than 90% already after two days post infection, whereas strain 3SWD1 never reached 40% of mortality in the entire time period of five days (Figure 5). Log-rank test analysis confirmed a statistically significant difference in survival among groups (Chi-square = 108, df = 5, *p* < 2 × 10^−16^). Notably, strains harbouring the *tviBCDE* and *vexABCDE* operons (1SBR104, 5SBR103 and 5SBR282) exhibited significantly greater virulence than strain 3SWD1, which lacked these operons (Figure 5). These loci are responsible for the synthesis and export, respectively, of the Typhi-specific Vi capsular antigen and the *pil* locus involved in type IV pilus formation [51]. In the newly sequenced genomes, these loci were in the chromosome (Figure 6). All four genomes carried a complete *csgABCDEFG* operon and five out of nine genes of the complete *fim* gene cluster previously described in a cytotoxic *C. braakii* isolated from a patient with chronic gastritis (Figure 4) [16]. In particular, the core part of the *fim* cluster was detected, including genes *fimF*, *fimH*, *fimD*, *fimC*, and *fimI*, whereas the regulatory genes *fimW*, *fimY* and *fimZ* upstream of the *fimF* gene, as well as the *fimA* gene downstream of the *fimI* gene, were missing in all *C. braakii* of the present study. The *csg* and *fim* gene clusters encode for fimbriae and pili involved in bacterial adhesion.

The higher pathogenicity of *C. braakii* strains carrying *tvi*BCDE and *vex*ABCDE operons is reinforced by the following observation. Comparing publicly available genomes of *C. braakii*, 103 out of 110 human genomes of clinical relevance carried complete versions of the two operons, along with only 60 out of 110 environmental genomes (Table S3). Statistical analyses suggested that the prevalence of both operons was significantly higher in publicly available human isolates (93.6%) compared to environmental ones (54.5%) (χ^2^ = 41.77, df = 1, *p* = 1.03 × 10^−10^). Considering a *p*-value threshold of 0.001, no statistically significant difference was observed for the presence of the *csg* gene cluster, which was detected in 109/110 (99.1%) human isolates and 101/110 (91.8%) environmental isolates (Fisher’s exact test: *p* = 0.0188, OR = 9.64, 95% CI: 1.30–428.68). Although its prevalence appeared higher in human isolates, this difference did not meet the pre-defined significance threshold.

Additionally, no statistically significant differences were observed among genomes of different sources in relation to complete *fim* (110/110 human genomes vs. 107/110 environmental genomes, *p* = 0.2466, OR = Inf, 95% CI: 0.415–Inf). Moreover, few genomes carried the complete *T6SS* gene cluster (4/110 human genomes; 1/110 environmental genomes; 5/32 animal genomes; and 0/85 food genomes). The results suggest that the *fim*, *csg* and *T6SS* gene clusters are not representative as key virulence markers.

## 4. Discussion

Among the *Enterobacteriaceae* previously collected from 1170 food samples, 20 isolates were confirmed as *C. braakii* and 1 as *C. freundii* in the present study. These isolates were collected from fermented food of animal origin and the processing environment in two artisanal facilities producing soft cheese and salami, respectively. Artisanal productions are perceived as more genuine by consumers; however, the reduced automation in artisanal plants is associated with a greater challenge in the control of production parameters and hygiene of food products [23,24]. Selected isolates were erroneously identified as belonging to *C. freundii* and *Klebsiella* spp. by biochemical tests and were reassigned as belonging to *C. braakii* and *C. freundii* after whole-genome sequencing-based analyses. These data confirm the unsuitability of current biotyping systems for the identification of a species like *C. braakii*, which often cross-reacts with other *Enterobacteriaceae* due to their proximity [6,7,8,9,10,11,20]. Of note is the fact that the manufacturer of the biotyping system reported the potential misidentification of taxa, such as *C. braakii*, not included in the manufacturer differential chart. The future increase in the application of whole-genome sequencing for the confirmation of bacterial pathogen species will potentially lead to an increase in the detection of *C. braakii*, which was recently described as accounting for 18% of all nosocomial *Citrobacter* infections from 2000 to 2022 [1].

Phylogenomic reconstruction gave interesting insights on the potential origin of contamination. Within the salami production, two clones of *C. braakii* were identified. These two clones were persistently isolated in consecutive batches in one year of sampling, namely CITRO1 and CITRO2. Regarding CITRO1, SNP calling analyses revealed the close genetic relationship of isolates collected from semi-finished food products and the processing environment of a specific area of the facility, namely the floor drains of the drying room. This observation suggests the potential role of floor drains as a source of contamination of the food product. These insights are of great relevance in hygiene management, suggesting the need to focus on good hygienic practices, specifically in this part of the facility. Sanitation and disinfection procedures of floor drains have been already described as potentially inappropriate, especially when floor drains are poorly accessible [52]. For these reasons, floor drains have been well known for many years as harbourage sites of foodborne pathogens [52,53,54]. *Citrobacter freundii* was described as the most prevalent species within carbapenem-producing Enterobacteriales isolated from drains of a hospital in Belgium [55]. The survival of *Citrobacter* spp. in soil, water and the processing environment is associated with the osmotic stress tolerance, biofilm formation and swimming mobility of these bacteria [56]. Regarding CITRO2, SNP calling analyses revealed the close genetic relationship between isolates collected from raw materials, semi-finished and finished products and the processing environment across the entire salami production chain, from the stuffing room to the ripening room, suggesting that contaminated raw materials could be a vehicle through which the *C. braakii* CITRO2 clone was introduced in the processing facility. After its introduction, the CITRO2 clone potentially spread within the different areas of the facility, persisting in consecutive batches over one year. The relevance of raw materials as potential sources of contamination for food of animal origin has extensively been reported [57,58]. Further studies on the animal reservoir are required to elucidate the source of contamination. In particular, it needs to be elucidated whether *C. braakii* colonizes pigs and can thus be ascribed as a zoonotic pathogen or whether it contaminates carcasses at slaughterhouses from the environment. In the literature, the colonization of the digestive tract of piglets by *C. freundii* has already been reported, as well as the detection of carbapenem-resistant *C. freundii* in faecal samples of slaughtered pigs [59,60].

*C. braakii* isolates of the present study showed a low burden of antimicrobial resistance to AmpC β-lactams and reduced susceptibility to fluoroquinolones. However, the molecular bases of these phenotypes (*bla_CMY_* and *qnrB*) were chromosomally located, indicating that these features are stable and are disseminated vertically from mother to daughter bacterial cells [46,47,48,49].

The phylogenetic reconstruction performed including publicly available genomes revealed the close genetic proximity of the CITRO2 clade from the salami production to publicly available genomes isolated from humans in China and The Netherlands. Similarly, the two *C. braakii* genomes from the soft cheese production showed high genetic relatedness with publicly available human genomes collected in the USA and Europe. Although these genetic distances were relatively low, suggesting a recent common ancestor, the strains were isolated in different countries and years, and no direct epidemiological link was available. Therefore, we interpret these results as indicative of phylogenetic proximity rather than recent clonal transmission. These findings confirm that some strains of *C. braakii* are of interest for public health and, most importantly, suggest that specifically foodborne *C. braakii* may act as opportunistic pathogens in humans. Pathogenic *C. braakii* of nosocomial importance has been already described, and *C. braakii* has already been isolated in food. What still needs to be addressed is whether food can act as a reservoir of pathogenic *C. braakii* [1,18,20,21].

The characterization of the virulome and pathogenicity assessment of *C. braakii* isolates of the present study address this issue. In particular, the data of the present study revealed the pathogenicity of foodborne *C. braakii* and suggested a higher pathogenicity of the strains carrying the *vexABCDE* and *tviBCDE* operons. The *tvi* and *vex* operons encode for the capsular polysaccharide Vi antigen [51,61]. In the literature, the *vex* genes (*vex*ABCDE) were reported as being associated with higher potential for the pathogenicity of *Salmonella* Typhi within humans [62,63]. Interestingly, the occurrence of the complete versions of the two operons in *C. braakii* was 94% (103/110) in human genomes of clinical relevance in comparison to only 55% (60/110) in environmental genomes, reinforcing the key role of the two operons in pathogenic *C. braakii*.

In *C. braakii*, little information is available on key virulence genes associated with clinical manifestation. A complete Type 6 secretion system and adhesion-related *fim* and *csg* curli fimbriae were predicted in cytotoxic *Citrobacter braakii* isolated from the stomach of a patient with chronic gastritis [16]. The T6SS was suggested to be involved in the pathogenicity of *C. braakii* by enhancing bacterial competition with other bacteria in the microbiome and facilitating the bacterial colonization of the host [64,65,66,67]. None of the newly sequenced genomes of *C. braakii* of the present study carried a complete T6SS gene cluster and few of the publicly available genomes included. A complete *csg* cluster was identified in all newly sequenced genomes, along with a truncated *fim* cluster, including a core set of five out of the nine genes of the complete cluster. In publicly available genomes of the present study, no significant difference was observed among the distribution of *fim* and *csg* in clusters based on an isolate source. The lack of the enrichment of *fim* and *csg* clusters and the low occurrence of the *T6SS* gene in human publicly available genomes suggests that these genes do not represent key virulence markers of *C. braakii*’s pathogenicity in humans.

The data determined in vivo are essential to reliably elucidate the true clinical potential of any virulence or pathogenic characterization predicted from the study of the whole genome. The larvae of *G. mellonella* has been extensively described as nonmammalian in an in vivo infection model, with significant advantages over mammalian models: (1) *G. mellonella* larvae have an innate immune system similar to that of mammalian cells; (2) they follow the FAIR principles of replacement, reduction and refinement; (3) at present, in Europe, no restrictions are in place for the use of invertebrates as in vivo models in experimental research; (4) they are cheap and relatively easy to handle; and (5) their efficacy in the assessment of the pathogenicity of different human bacterial pathogens has been described [68,69,70,71,72,73]. Along with its advantages, it is worth mentioning that *G. mellonella* also faces limitations as an in vivo infection model. Although possessing an innate immune system similar to mammalian cells, it lacks adaptive immunity, reinforcing the idea that *G. mellonella* cannot fully replace more complex animal models such as mice [74]. In the present study, the assessment with the in vivo infection model of *G. mellonella* provided the first indication of the potential association of *vex* and *tvi* gene clusters with higher pathogenicity in *C. braakii*. In fact, *C. braakii* isolates carrying the two operons showed higher pathogenicity than those lacking the two operons, with a mortality rate of *G. mellonella* of 90% vs. 40%. Further in vivo infection studies using mice are needed to confirm these findings.

## 5. Conclusions

*Citrobacter braakii* was identified in two artisanal productions of soft cheese and salami from semi-finished and finished products and from the processing area. In the salami production, WGS-based analyses revealed two persistent clones over a year of sampling, indicating ripening room drains and raw materials as contamination sources. The presence of potentially pathogenic *C. braakii* in food processing plants reinforce the importance of good hygienic practices, especially in artisanal productions, where the control of production parameters is challenging. The partial automation of production is additionally suggested in order to achieve better control of parameters and the reduced manipulation of food by workers. Research on the transmission pathways from food-producing animals, such as swine, to food of animal origin, such as salami, is required in order to understand the reasons for raw material contamination and to find effective mitigation strategies. Comparisons with human genomes of clinical relevance and pathogenicity assessments in the in vivo *Galleria mellonella* infection model pointed towards one clone and two clones of the salami and soft cheese productions, respectively, showing high genetic similarity with publicly available human clinical genomes and higher pathogenicity associated with *vex* and *tvi* gene clusters related to capsular polysaccharide (Vi antigen) production. Although non-conclusive, *G. mellonella*-based results suggest *vex*- and *tvi*-positive *C. braakii* strains as presumptive food safety hazards.

## Figures and Tables

**Table 1:** Species identification of bacterial isolates by biotyping and whole-genome sequencing.

Sample	Biotyping	WGS ^a^ Refseq	ANI ^b^ (%)	Food Production	Sample Origin	Batch	ST ^c^
5CP1581	*C. freundii*	*C. braakii*	99.08	Cheese	Finished product	5	984
6CP485A	*K. pneumoniae*	*C. freundii*	99.75	Cheese	Finished product	6	617
6CP11281B	*C. freundii*	*C. braakii*	99.10	Cheese	Finished product	6	905
1SBD4	*C. freundii*	*C. braakii*	99.07	Salami	Semi-finished product	1	1267
1SBR5	*C. freundii*	*C. braakii*	99.06	Salami	Semi-finished product	1	1267
1SBR104	*C. freundii*	*C. braakii*	99.05	Salami	Semi-finished product	1	1267
2SBD4	*C. freundii*	*C. braakii*	99.05	Salami	Semi-finished product	2	1267
2SBR2	*C. freundii*	*C. braakii*	99.07	Salami	Semi-finished product	2	1267
2SBR184	*C. freundii*	*C. braakii*	99.20	Salami	Semi-finished product	2	1117
3SWD1	*C. freundii*	*C. braakii*	99.22	Salami	Environment	3	1268
3SBR5	*C. freundii*	*C. braakii*	99.20	Salami	Semi-finished product	3	1117
4SBD2	*C. freundii*	*C. braakii*	99.04	Salami	Semi-finished product	4	1267
4SWD3	*C. freundii*	*C. braakii*	99.04	Salami	Environment	4	1267
5SWD1	*C. freundii*	*C. braakii*	99.22	Salami	Environment	5	1268
5SBR3	*C. freundii*	*C. braakii*	99.20	Salami	Semi-finished product	5	1117
5SBR103	*C. freundii*	*C. braakii*	99.22	Salami	Semi-finished product	5	1269
5SBR183	*C. freundii*	*C. braakii*	99.20	Salami	Semi-finished product	5	1117
5SBR282	*C. freundii*	*C. braakii*	99.40	Salami	Semi-finished product	5	1117
6MB1	*C. freundii*	*C. braakii*	99.20	Salami	Raw material	6	1117
6STM5	*K. oxytoca*	*C. braakii*	99.20	Salami	Environment	6	1117
6SBR1	*C. freundii*	*C. braakii*	99.22	Salami	Semi-finished product	6	1117

Notes: ^a^ WGS = whole-genome sequencing. ^b^ ANI = Average Nucleotide Identity. ^c^ ST = sequence type.

## Data Availability

The paired-end reads included in this study have been deposited in the European Nucleotide Archive (ENA) at EMBL-EBI under accession number PRJEB82867 (https://www.ebi.ac.uk/ena/browser/view/PRJEB82867, accessed on 27 November 2024).

## Supplementary Materials

The following supporting information can be downloaded at: https://www.mdpi.com/article/10.3390/foods14111887/s1, Table S1: Publicly available genomes of human, food, animal and environmental origin included in this study and related assembly statistics. Table S2: Matrix of antimicrobial resistance genes of publicly available and newly sequenced *Citrobacter* spp. Table S3: Matrix of virulence genes of publicly available and newly sequenced *Citrobacter* spp. Table S4: Quality statistics of de novo assemblies of 20 newly sequenced *Citrobacter* spp. genomes. Table S5: Multi-locus sequence types of newly sequenced *C. braakii* genomes. Table S6: Pairwise SNP distance matrix of newly sequenced and publicly available *Citrobacter* spp. genomes included in this study. Table S7: Antimicrobial susceptibility testing of newly sequenced *C. braakii* genomes. The table presents the minimum inhibitory concentrations (MICs) for each tested strain against a panel of antimicrobial agents. MIC values are reported in μg/mL. Table S8: ampR gene alignment between 3SWD1, 5SWD1 and 6STM5 *C. braakii* strains and *C. freundii* reference strain (NZ_CP033744.1).
