# Supplementary material for: Citrobacter braakii Isolated from Salami and Soft Cheese: An Emerging Food Safety Hazard?
Source: Foods. 2025 May 26;14(11):1887. doi: 10.3390/foods14111887 (PMC12154454; doi:10.3390/foods14111887)
Supplement: Supplementary file 1 [file foods-14-01887-s001.zip › supplementary files/Table S4.docx]

Table S4 - Quality statistics of *de novo* assemblies of 20 newly sequenced *Citrobacter spp.* genomes

| Genome | N. contigs | Largest contig | Total lenght (bp) | N50 | %GC |
| --- | --- | --- | --- | --- | --- |
| 5CP1581 | 139 | 847099 | 5590886 | 310045 | 52.07% |
| 6CP485A | 156 | 231340 | 5156793 | 99247 | 51.00% |
| 6CP11281B | 232 | 405423 | 5627446 | 145919 | 52.00% |
| 1SBD4 | 93 | 686363 | 5174450 | 230516 | 52.08% |
| 1SBR5 | 97 | 508462 | 5173660 | 227985 | 52.10% |
| 1SBR104 | 113 | 686363 | 5222301 | 227985 | 52.20% |
| 2SBD4 | 111 | 686363 | 5221962 | 230516 | 51.87% |
| 2SBR2 | 96 | 686363 | 5174320 | 230516 | 52.05% |
| 2SBR184 | 38 | 753115 | 4754737 | 443851 | 52.08% |
| 3SWD1 | 138 | 387798 | 5091654 | 116376 | 52.01% |
| 3SBR5 | 39 | 840855 | 4845062 | 528096 | 51.98% |
| 4SBD2 | 107 | 686336 | 5222304 | 230516 | 51.85% |
| 4SWD3 | 117 | 738175 | 5235364 | 262586 | 51.92% |
| 5SWD1 | 135 | 387798 | 5091852 | 102967 | 52.07% |
| 5SBR3 | 46 | 753114 | 4844144 | 443851 | 52.20% |
| 5SBR103 | 65 | 2641110 | 5076362 | 2641110 | 52.14% |
| 5SBR183 | 38 | 753115 | 4754739 | 443851 | 52.18% |
| 5SBR282 | 44 | 722259 | 4843913 | 528095 | 52.21% |
| 6MB1 | 39 | 753115 | 4754736 | 443851 | 52.06% |
| 6STM5 | 39 | 753114 | 4755044 | 443851 | 52.00% |
| 6SBR1 | 38 | 753115 | 4754737 | 443851 | 52.11% |
